# Associations of varicose veins with cerebrospinal fluid biomarkers of Alzheimer’s disease pathologies in adults without dementia: the CABLE study

**DOI:** 10.3389/fnagi.2025.1502154

**Published:** 2025-03-04

**Authors:** Min Liu, Li-Yun Ma, Qiong-Yao Li, Liang-Yu Huang, He-Ying Hu, Lan Tan, Hao Hu

**Affiliations:** ^1^Department of Neurology, Qingdao Municipal Hospital, Dalian Medical University, Qingdao, China; ^2^Department of Neurology, Qingdao Municipal Hospital, Qingdao University, Qingdao, China

**Keywords:** Alzheimer’s disease, dementia, varicose veins, biomarkers, inflammation

## Abstract

**Background:**

Previous studies have found a correlation between varicose veins (VVs) and cognitive decline, and individuals with VVs have a higher prevalence of Alzheimer’s disease (AD). However, the associations between VVs and the core pathologies of AD have not yet been investigated. The research was designed to analyze the relationships between VVs and cerebrospinal fluid (CSF) biomarkers of AD pathologies.

**Methods:**

We included 1,298 participants from the Chinese Alzheimer’s Biomarker and LifestylE (CABLE) database without dementia. Multiple linear regression (MLR) model was applied to assess the relationships between the VVs and CSF AD biomarkers. Then, we conducted subgroup analyses according to age, gender, education levels and *apolipoprotein E genotype ε4 (APOE-ε4)* carrier status. Additionally, mediation effects were assessed using causal mediation analyses with 10,000 bootstrapped iterations.

**Results:**

In total subjects, VVs had negative correlations with CSF Aβ_42_ (β = −0.157, *p* = 0.038) and CSF Aβ_42_/Aβ_40_ ratio (β = −0.272, *p* < 0.001), as well as positive correlations with CSF Aβ_40_ (β = 0.170, *p* = 0.024), CSF p-tau (β = 0.192, *p* = 0.008), CSF t-tau/Aβ_42_ ratio (β = 0.190, *p* = 0.011), and CSF p-tau/Aβ_42_ ratio (β = 0.248, *p* = 0.001), after adjusting for age, sex, education levels and *APOE-ε4* carrier status. Subgroup analyses demonstrated that the relations between VVs and CSF AD biomarkers were more significant in female, mid-life adults (40–65 years), less-educated individuals and *APOE-ε4* non-carriers. Moreover, CSF Aβ_42_/Aβ_40_ ratio might be a partial mediator of the association between VVs and p-tau pathology.

**Conclusion:**

Our study found correlations between VVs and CSF AD biomarkers, suggesting that VVs may be a potential risk factor for the development of AD.

## Introduction

As the major type of dementia, Alzheimer’s disease (AD) has emerged as one of the most costly, deadly and burdening diseases in recent decades. The main pathological changes of AD consist of neurodegeneration as well as the accumulation of toxic amyloid-beta (Aβ) and hyperphosphorylated tau (Alzheimers Dementia, 2023; Scheltens et al., 2021). Due to the complex pathophysiological changes in AD, a complete understanding of its pathology is lacking. As a result, AD cannot currently be cured by reversing its progression (Liu et al., 2023). However, there is potential for prevention by reducing harmful risk factors. Therefore, exploring these risk factors associated with AD and intervening at an early stage is essential.

Cardiovascular diseases have been extensively studied for their association with AD, and some vascular diseases have been identified as risk factors for AD (Cortes-Canteli and Iadecola, 2020; Tublin et al., 2019). For instance, hypertension has been recognized as an independent risk factor for AD (Tublin et al., 2019), and *The Lancet* confirmed that midlife hypertension is a risk factor for AD, with a relative risk (RR) of 1.6, 95% confidence intervals (CI) 1.2–2.2 (Livingston et al., 2020). Atherosclerosis, including both intracranial and extracranial arterial involvement, has been found to have a correlation with AD (Cortes-Canteli and Iadecola, 2020; Hofman et al., 1997). Aortic stiffening has also been shown in study to have an association with cognitive impairment and the development of dementia (Pase et al., 2016). Additionally, a higher burden of cerebral small vessel disease (CSVD) has also been associated with cognitive decline and increased risk of developing AD (Sun et al., 2023). Importantly, an autopsy-based neuropathological study found that 80% of patients diagnosed with AD and no evidence of mixed (vascular) dementia had vascular pathology (Sweeney et al., 2019). This suggested that vascular factors play a crucial role in AD, the extent of which may not yet be fully understood. Although the relationship between vascular diseases and cognition, as well as AD has been studied, the majority of research has focused on arterial system diseases, while venous system diseases have often been ignored, particularly the varicose veins (VVs) that are common in population. Until now, the relationships of VVs with cognition and AD incidence have been less studied. In a cohort, both cross-sectional and longitudinal studies indicated that VVs were associated with decreased Mini-Mental State Examination (MMSE) and Montreal Cognitive Assessment (MoCA) scores (Kujawski et al., 2018; Kujawski et al., 2021). Recently, a large cohort study showed that patients with VVs had an elevated incidence of developing AD (Cheng, 2022). These studies suggest a potential correlation between VVs and cognition as well as AD, but relevant research is scarce, which has inspired our interest in exploring the relationship between VVs and AD.

With advances in biomarkers, those reflecting AD core pathology, including Aβ_42_, phosphorylated tau (p-tau) and total tau (t-tau) in cerebrospinal fluid (CSF), can be detected many years before the onset of clinical symptoms (Bateman et al., 2012; Grontvedt et al., 2018). Additionally, the latest 2024 Alzheimer’s Association (AA) criteria have incorporated these CSF core biomarkers into the updated ATX research framework, which aims to capture the pathological changes of AD (Jack et al., 2024). Thus, exploring the relationships between AD risk factors and biomarkers can provide valuable insights into their associations with AD and its underlying mechanisms. It is worth noting that there is currently a lack of research into the relationships between VVs and CSF AD biomarkers, therefore, this study aims to assess whether the presence of VVs is correlated with CSF AD biomarkers, which will add to the evidence of the complex interaction between vascular disease and AD pathologies.

## Materials and methods

### The CABLE database

The present study used data from the ongoing Chinese Alzheimer’s Biomarker and LifestylE (CABLE) database, a large-scale investigation into AD risk factors and CSF biomarkers in the northern Chinese Han population since 2017. The CABLE sought to identify genetic and environmental modifiers of AD biomarkers, aiming to enhance AD prevention and early detection in the non-demented northern Han Chinese population. All participants were recruited from Qingdao Municipal Hospital, Shandong Province, China. The CABLE study was ethically approved by the Institutional Ethics Committee of Qingdao Municipal Hospital, following the Declaration of Helsinki, with informed consent obtained from all subjects or their proxies.

### Study participants

All CABLE participants, aged 40–90, were Han Chinese, with exclusion criteria encompassing: (1) central nervous system infection, head trauma, neurodegenerative diseases other than AD (e.g., Parkinson’s Disease, epilepsy), or other major neurological disorders; (2) major psychological disorders; (3) severe systemic diseases (e.g., malignant tumors); (4) family history of genetic diseases. All participants included in the study underwent cognitive, clinical and neuropsychological assessments, together with their biosamples (blood and CSF) collected by physicians with uniformed training. The Chinese-Modified Mini-Mental State Examination (CM-MMSE) was used to evaluate global cognition at baseline. The score on the CM-MMSE spans from 0 to 30, with elevated scores reflecting superior general cognitive functioning. Medical histories of all the participants were obtained from the electronic medical record (EMR) system. Eligible subjects were divided into VVs group and non-VVs group.

A total of 1,982 participants in the CABLE without dementia provided available covariate data [age, gender, education levels, *apolipoprotein E genotype ε4 (APOE-ε4)* carrier status and so on]. We excluded individuals without CSF biomarker data and those whose data were beyond 4 standard deviations (SD). Finally, our cross-sectional study included 1,298 participants.

### Assessments of CSF AD biomarkers

CSF specimens were obtained through a lumbar puncture after an overnight fast. Within 2 h of collection, these samples were centrifuged at 2,000 g at room temperature for 10 min to remove the cells and other insoluble substances. The CSF samples were preserved in refrigerators at −*80*°C for subsequent analysis, with each analysis limited to one tube per sample to minimize freeze-thaw cycles (maximal two, preferably one). Baseline CSF Aβ_42_, Aβ_40_, tau, and p-tau were determined with the ELISA kit [Innotest β-AMYLOID (1-42), β-AMYLOID (1-40), hTAU-Ag, and PHOSPHO-TAU (181p); Fujirebio, Ghent, Belgium] on the microplate reader (Thermo Scientific™ Multiskan™ MK3). Random distribution and duplicate measurements were ensured across plates, all antibodies and plates were sourced from the same batch to reduce batch-to-batch variability. Experienced technicians, who were unaware of the information provided by the participants, conducted the experiments, maintained intra-batch variable coefficient (CV) < 5% and inter-batch CV < 15%. Quality control evaluations confirmed CSF biomarker levels were independent of storage duration, collection time, intra-batch, and inter-batch CVs.

### *APOE-ε4* genotyping

After collection, fasting blood specimens underwent centrifugation at 2,000 g for 10 min and were stored at −*20*°C, with a maximum of two freeze-thaw cycles. Deoxyribonucleic acid (DNA) extraction from fasting blood utilized the QIAamp^®^ DNA Blood Mini Kit (250). Genotyping for *APOE-ε4* status at loci rs7421 and rs429358 was performed using restriction fragment length polymorphism (RFLP) technology. Participants were then categorized into *APOE-ε4* non-carriers (lacking the *APOE-ε4* allele) and *APOE-ε4* carriers (possessing at least one copy of the *APOE-ε4* allele).

### Covariates

Basic covariates included gender (male or female), age (continuous), education levels (continuous) and *APOE-ε4* carrier status (carriers or non-carriers). In addition, lifestyle behaviors and clinical complications were identified as potential confounders. Data on lifestyle behaviors were obtained via a well-structured questionnaire, including body mass index (BMI, continuous), present or previous drinkers (yes or no), present or previous smokers (yes or no), along with regular physical activity (yes or no). Information on comorbidities was obtained from diagnoses or medical histories documented in the EMR system, including histories of hypertension, diabetes, stroke, hyperlipemia and coronary heart disease (CHD).

### Statistical analysis

We screened the data on CSF biomarkers and excluded the outliers (outside 4 SDs). Since CSF biomarkers did not conform to a standard distribution (Shapiro-Wilk test in R, *p* < 0.0001), they were normalized by Box-Cox transformation through the “car” package of R software and standardized by z-scale (Chen and Pounds, 1998). Chi-squared test (for categorical variables) and *t*-test or Mann-Whitney U test (for continuous variables) were employed to compare demographic characteristics between VVs group and non-VVs group. Continuous and categorical variables were presented as mean ± SD and proportion (%), respectively. After adjusting for different covariates, multiple linear regression (MLR) was conducted to explore the correlation between VVs and each CSF biomarker. The prevalence of AD is on the rise as the population ages, and the upper limit of onset age for early-onset AD has been suggested to be 65 years old (Hou et al., 2019). Additionally, the age of 65 is a universally acknowledged boundary for the definition of old age (Rossor et al., 2010). We used the threshold of 65 years old to divide the participants into mid-life group (≤ 65 years old, *n* = 797, 61.4%) and late-life group (> 65 years of age, *n* = 501, 38.6%). As the average education levels for all participants was around 9 years, the participants were separated into less-educated (≤ 9 years of education) and higher-educated (> 9 years of education) groups. We performed subgroup analyses based on gender (male and female), age (≤ 65 years and > 65 years), education levels (≤ 9 years and > 9 years) along with *APOE-ε4* carrier status (carriers or non-carriers).

To assess whether Aβ pathology mediated the link between VVs and p-tau pathology, we fitted an MLR model using the Baron and Kenny method (Baron and Kenny, 1986). For the three equations, (1) Aβ pathology (the mediator) was regressed on VVs (the independent variable), (2) p-tau pathology (the dependent variable) was regressed on the independent variable, and (3) the dependent variable was regressed on both the mediator and the independent variable. Whereafter, we assessed the attenuation or indirect effect, determining significance through 10,000 bootstrapped iterations. In this model, every pathway was corrected for *APOE-ε4* carrier status, education levels, gender and age.

A two-sided *P*-value of < 0.05 was regarded as significant. Statistical analyses and graph generation were conducted using R Studio software (version 4.2.2).

## Results

### Basic characteristics of the study participants

**FIGURE 1:**
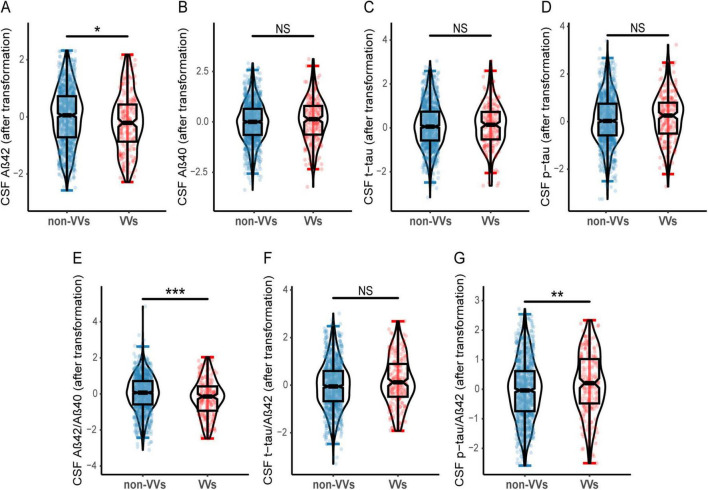
Differences of CSF AD biomarkers in VVs and non-VVs groups. Participants with VVs had lower CSF Aβ_42_ level **(A)**, as well as lower CSF Aβ_42_/Aβ_40_ ratio **(E)** compared to these without VVs. Participants with VVs had higher CSF p-tau/Aβ_42_ ratio **(G)** compared to these without VVs. And there were no significant differences in CSF Aβ_40_ **(B)**, t-tau **(C)**, p-tau **(D)**, t-tau/Aβ_42_ ratio **(F)** between the VVs and non-VVs groups. VVs, Varicose veins; CSF, cerebrospinal fluid; Aβ, amyloid-β; t-tau, total tau; p-tau, phosphorylated tau; NS, Not Significant. *p < 0.05, **p < 0.01, ***p < 0.001.

A total of 1,298 participants were included from the CABLE database, including 209 (16.1%) with VVs. Demographic and clinical characteristics were described in Table 1, including age (range, 40–88 years; mean age, 62.09 ± 10.71 years), gender (female proportion, 41.4%), average education levels (9.55 years), proportion of *APOE-ε4* carriers (15.5%), and cognitive scores (mean CM-MMSE scores, 27.76 ± 2.21; mean MoCA scores, 23.08 ± 4.47). Gender, education levels and *APOE-ε4* carrier status did not differ significantly between the two groups. In contrast to the non-VVs group, the VVs group showed lower CSF Aβ_42_, Aβ_42_/Aβ_40_ ratio, along with a higher CSF p-tau/Aβ_42_ ratio (Figure 1).

**TABLE 1:** Characteristics of participants.

Variables	Total	Non-VVs	VVs	*P*-value
*N*	1,298	1,089	209	–
Age, year, mean (SD)	62.09 (10.17)	62.63 (10.44)	59.31 (8.11)	**< 0.001**
Female gender, *N* (%)	538 (41.4)	440 (40.4)	98 (46.9)	0.096
Education, years, mean (SD)	9.55 (4.35)	9.47 (4.39)	9.96 (4.16)	0.111
Positive *APOE-ε4* carrier status, *N* (%)	201 (15.5)	165 (15.2)	36 (17.2)	0.513
CM-MMSE scores, mean (SD)	27.76 (2.21)	27.71 (2.21)	28.01 (2.18)	**0.027**
MoCA scores, mean (SD)	23.08 (4.47)	22.96 (4.54)	23.69 (4.06)	0.088
BMI, mean (SD)	25.56 (3.62)	25.57 (3.69)	25.52 (3.23)	0.893
Smoking (yes, %)	372 (28.7)	316 (29.1)	56 (26.9)	0.582
Alcohol (yes, %)	384 (29.6)	318 (29.3)	66 (31.6)	0.554
Physical exercise (yes, %)	563 (43.5)	453 (41.8)	110 (52.9)	**0.004**
**Comorbidities (yes, %)**				
Hypertension	492 (38.0)	426 (39.2)	66 (31.6)	**0.046**
Diabetes	184 (14.3)	164 (15.1)	20 (9.6)	**0.048**
CHD	162 (12.5)	149 (13.7)	13 (6.2)	**0.004**
Stroke	48 (3.7)	45 (4.1)	3 (1.4)	0.090
Hyperlipemia	73 (5.6)	62 (5.7)	11 (5.3)	0.927
**CSF AD biomarkers and rations, mean (SD)**				
Aβ_42_ (pg/mL)	334.10 (212.48)	339.50 (212.08)	306.01 (212.89)	**0.016**
Aβ_40_ (pg/mL)	6581.33 (2763.25)	6523.12 (2721.54)	6884.67 (2959.51)	0.134
t-tau (pg/mL)	195.24 (82.75)	195.49 (82.85)	193.98 (82.39)	0.730
p-tau (pg/mL)	43.58 (13.49)	43.38 (13.50)	44.62 (13.41)	0.190
Aβ_42_/Aβ_40_ ratio	0.056 (0.047)	0.058 (0.049)	0.048 (0.031)	**< 0.001**
t-tau/Aβ_42_ ratio	0.87 (0.79)	0.85 (0.77)	0.99 (0.88)	0.057
p-tau/Aβ_42_ ratio	0.20 (0.17)	0.19 (0.16)	0.23 (0.18)	**0.002**

Bold indicated that differences in non-VVs group and VVs group were significant. Significant differences in non-VVs group and VVs group were used by the *t*-test or Mann-Whitney U test (for continuous variables) and Chi-squared test (for categorical variables). *APOE-ε4*, *apolipoprotein E genotype ε4*; CM-MMSE, China-Modified Mini-Mental State Examination; MoCA, Montreal Cognitive Assessment; CSF, cerebrospinal fluid; AD, Alzheimer’s disease; Aβ, amyloid-β; t-tau, total tau; p-tau, phosphorylated tau; BMI, body mass index; CHD, coronary heart disease; SD, standard deviation.

### Correlations of VVs with CSF AD biomarkers

The study investigated the connections between VVs and CSF AD biomarkers using an MLR model, and the results were presented in Table 2. In model 1, after adjustment for basic covariates (age, gender, *APOE-ε4* carrier status and education levels), VVs showed significant associations with reduced CSF Aβ_42_ (β = −0.157, *p* = 0.038), and Aβ_42_/Aβ_40_ ratio (β = −0.272, *p* < 0.001), along with elevated CSF Aβ_40_ (β = 0.170, *p* = 0.024), p-tau (β = 0.192, *p* = 0.008), t-tau/Aβ_42_ ratio (β = 0.190, *p* = 0.011) and p-tau/Aβ_42_ ratio (β = 0.248, *p* = 0.001). However, no significant association was observed between VVs and CSF t-tau (β = 0.082, *p* = 0.254). Lifestyle factors (including BMI, smoking, alcohol consumption, and physical exercise), as well as histories of hypertension, diabetes and other diseases have been reported to influence the onset and development of AD (Pasqualetti et al., 2022; Silva et al., 2019). Therefore, we additionally adjusted for various covariates to perform sensitivity analyses. In model 2, we adjusted for basic covariates and lifestyle factors including BMI, smoking, alcohol consumption and physical exercise. In model 3, basic covariates and medical history including CHD, stroke, hyperlipemia, hypertension and diabetes were adjusted. In model 4, in addition to basic covariates, lifestyle factors, clinical comorbidities and CM-MMSE scores were additionally adjusted. The correlations between VVs and CSF AD biomarkers in model 2 to model 4 were similar to the results in model 1.

**TABLE 2:** Associations of VVs with CSF AD biomarkers.

	Aβ_42_		Aβ_40_		t-tau		p-tau		Aβ_42_/Aβ_40_		t-tau/Aβ_42_		p-tau/Aβ_42_	
	**β**	***P***	**β**	***P***	**β**	***P***	**β**	***P***	**β**	***P***	**β**	***P***	**β**	***P***
Model 1	−0.1567	**0.0384**	0.1699	**0.0240**	0.0818	0.2540	0.1923	**0.0082**	−0.2724	**< 0.001**	0.1900	**0.0113**	0.2483	**0.0010**
Model 2	−0.1665	**0.0306**	0.1915	**0.0121**	0.1107	0.1288	0.2100	**0.0046**	−0.2970	**< 0.001**	0.2136	**0.0054**	0.2641	**< 0.001**
Model 3	−0.1558	**0.0396**	0.1732	**0.0220**	0.0808	0.2620	0.1909	**0.0092**	−0.2727	**< 0.001**	0.1885	**0.0123**	0.2470	**0.0011**
Model 4	−0.1631	**0.0344**	0.1932	**0.0119**	0.1110	0.1297	0.2107	**0.0047**	−0.2935	**< 0.001**	0.2103	**0.0061**	0.2612	**< 0.001**

Model 1: multiple linear regression models after adjusted for age, gender, education levels, *APOE-ε4* status. Model 2: multiple linear regression models after adjusted for age, gender, education levels, *APOE-ε4* status, BMI, cigarette (yes versus no), alcohol (yes versus no), and physical exercise (yes versus no). Model 3: multiple linear regression models after adjusted for age, gender, education levels, *APOE-ε4* status, hypertension (yes versus no), diabetes (yes versus no), stroke (yes versus no), hyperlipemia (yes versus no) and CHD (yes versus no). Model 4: multiple linear regression models after adjusted for age, gender, education levels, *APOE-ε4* status, BMI, cigarette (yes versus no), alcohol (yes versus no), and physical exercise (yes versus no), hypertension (yes versus no), diabetes (yes versus no), stroke (yes versus no), hyperlipemia (yes versus no), CHD (yes versus no) and CM-MMSE scores. VVs, Varicose veins; CSF, cerebrospinal fluid; AD, Alzheimer’s disease; Aβ, amyloid-β; t-tau, total tau; p-tau, phosphorylated tau; *APOE-ε4*, *apolipoprotein E genotype ε4*; BMI, body mass index; CHD, coronary heart disease; CM-MMSE, China-Modified Mini-Mental State Examination. Bold values indicated statistically significant results.

### Subgroup analyses

**FIGURE 2:**
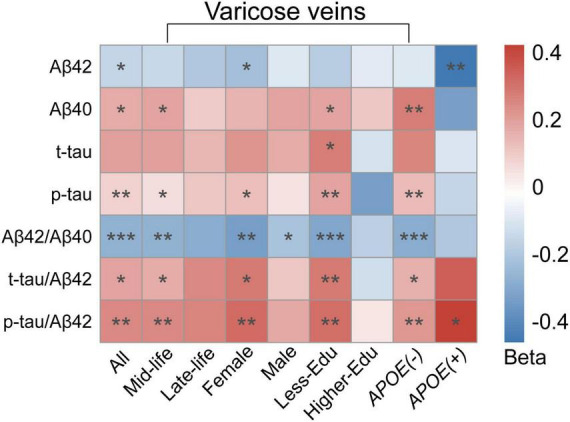
Heatmap for subgroup analyses of the association between VVs and CSF AD biomarkers. Multiple linear regression models were employed with adjustment for age, gender, education levels, *APOE-ε4* carrier status. In the subgroup analyses, the association between VVs and CSF AD biomarkers was stronger correlated in mid-life individuals, female, individuals with less education and individuals without *APOE-ε4* gene. Colors represented beta-estimates, asterisks indicated statistical significance (*p < 0.05, **p < 0.01, ***p < 0.001). VVs, Varicose veins; *APOE-ε4*, *apolipoprotein E genotype ε4*; CSF, cerebrospinal fluid; AD, Alzheimer’s disease; Aβ, amyloid-β; t-tau, total tau; p-tau, phosphorylated tau.

In our subgroup analyses, we observed that the correlations between VVs and CSF AD biomarkers were more significant in mid-life, female, *APOE-ε4* non-carriers and less-educated subgroups. In mid-life individuals, VVs were correlated to CSF Aβ_40_ (β = 0.184, *p* = 0.036), p-tau (β = 0.193, *p* = 0.024), Aβ_42_/Aβ_40_ (β = −0.270, *p* = 0.002), t-tau/Aβ_42_ (β = 0.169, *p* = 0.048) and p-tau/Aβ_42_ (β = 0.248, *p* = 0.004), while in the late-life individuals, VVs were not associated with any biomarkers. In females, VVs were correlated to CSF Aβ_42_ (β = −0.228, *p* = 0.046), p-tau (β = 0.219, *p* = 0.050), Aβ_42_/Aβ_40_ (β = −0.339, *p* = 0.003), t-tau/Aβ_42_ (β = 0.287, *p* = 0.012) and p-tau/Aβ_42_ (β = 0.322, *p* = 0.005). However, in the male subgroup, VVs were only related to CSF Aβ_42_/Aβ_40_ ratio (β = −0.217, *p* = 0.032). In the *APOE-ε4* non-carriers, VVs were strongly associated with CSF Aβ_40_ (β = 0.272, *p* = 0.001), p-tau (β = 0.254, *p* = 0.002), Aβ_42_/Aβ_40_ (β = −0.291, *p* < 0.001), t-tau/Aβ_42_ (β = 0.163, *p* = 0.049) and p-tau/Aβ_42_ (β = 0.217, *p* = 0.009). In the *APOE-ε4* carriers, VVs were only associated with CSF Aβ_42_ (β = −0.463, *p* = 0.009) and p-tau/Aβ_42_ ratio (β = 0.422, *p* = 0.018). In less-educated subgroup, VVs were associated with CSF Aβ_40_ (β = 0.225, *p* = 0.022), t-tau (β = 0.222, *p* = 0.019), p-tau (β = 0.270, *p* = 0.005), Aβ_42_/Aβ_40_ (β = −0.329, *p* < 0.001), t-tau/Aβ_42_ (β = 0.293, *p* = 0.003) and p-tau/Aβ_42_ (β = 0.296, *p* = 0.003), while there were no association in the higher-educated subgroup (Figure 2 and Supplementary Table 1).

### Mediation analyses

**FIGURE 3:**
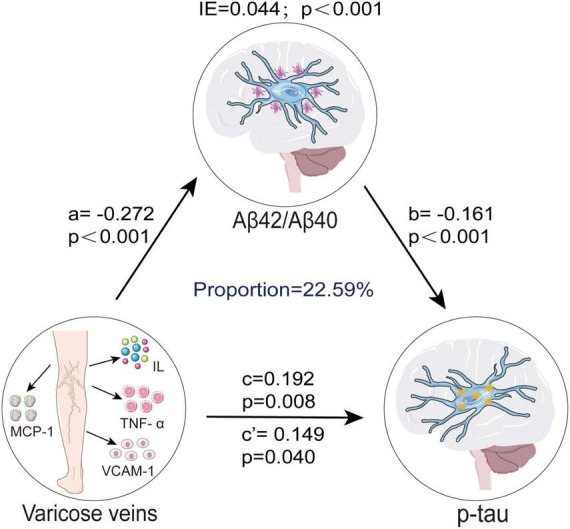
CSF Aβ_42_/Aβ_40_ ratio mediated association between VVs and p-tau. Causal mediation analyses with 10,000 bootstrapped iterations were used to examine the mediation effects of Aβ_42_/Aβ_40_ on p-tau. Each model path was adjusted for age, gender, education levels, and *APOE-ε4* carrier status. In this model, a represents the effect of VVs on CSF Aβ_42_/Aβ_40_ ratio, while b represents the effect of CSF Aβ_42_/Aβ_40_ ratio on p-tau pathology. c denotes the total effect of VVs on p-tau pathology before accounting for the mediator, whereas c’ indicates the direct effect of VVs on p-tau pathology after adjusting for CSF Aβ_42_/Aβ_40_ ratio. The IE, calculated as a × b, quantifies the mediation effect, reflecting the extent to which CSF Aβ_42_/Aβ_40_ ratio mediates the association between VVs and p-tau pathology. VVs, Varicose veins; IE, indirect effect; *APOE-ε4, apolipoprotein E genotype ε4*; CSF, cerebrospinal fluid; Aβ, amyloid-β; p-tau, phosphorylated tau; MCP, monocyte chemoattractant protein; IL, interleukin; TNF, tumor necrosis factor; VCAM, vascular cell adhesion protein.

Based on the above findings, we further investigated whether CSF Aβ pathology mediated the association between VVs and p-tau pathology. We did not analyze the mediating effects of t-tau pathology due to the absence of a significant association with VVs. Mediation analysis revealed that CSF Aβ_42_/Aβ_40_ ratio significantly mediated the relationship between VVs and p-tau pathology. Both the direct effect (β = 0.149, *p* = 0.040) of VVs on p-tau pathology and the indirect effect (β = 0.044, *p* < 0.001) through CSF Aβ_42_/Aβ_40_ ratio were statistically significant, indicating that CSF Aβ_42_/Aβ_40_ ratio partially mediated this association, with a mediating proportion of 22.59% (Figure 3).

## Discussion

This population-based study was the first to explore the relations between VVs and CSF AD biomarkers in the non-demented population. Our results clearly presented that the VVs were correlated with reduced CSF Aβ_42_ and Aβ_42_/Aβ_40_ ratio, along with increased CSF Aβ_40_, p-tau, t-tau/Aβ_42_ ratio and p-tau/Aβ_42_ ratio. However, we found no evidence for a significant correlation between VVs and CSF t-tau. Subgroup analysis indicated that the correlations between VVs and CSF AD biomarkers were more significant in mid-life, female, *APOE-ε4* non-carriers and less-educated individuals. Mediation analysis suggested that CSF Aβ_42_/Aβ_40_ ratio might mediate the correlation between VVs and p-tau pathology.

To our knowledge, this study was the first to examine the correlations between VVs and CSF AD biomarkers in non-demented participants. Although there have been limited studies on the relationship between VVs and cognition or the occurrence of AD, they have also provided some indirect evidence for the association between VVs and AD. We found that VVs were mainly associated with Aβ pathology, and the association with tau pathology was only observed in the association with p-tau. This discovery has significant implications for considering VVs as a potential target for interventions and treatments of early AD pathology.

The specific mechanisms by which VVs contribute to the development of pathological changes in AD are unclear. One possible mechanism is inflammatory pathways. Some studies have shown that patients with VVs have a raised level of inflammatory markers, including monocyte chemoattractant protein-1 (MCP-1), interleukin (IL)-6, and vascular cell adhesion protein-1 (VCAM-1) compared to healthy controls (Arase et al., 2019; Mikuła-Pietrasik et al., 2016). Additionally, a recent study showed that patients with venous ulcers of the lower limbs had higher expression of pro-inflammatory cytokines such as tumor necrosis factor (TNF)-α and IL-1α in peripheral blood mononuclear cells than controls (Filkor et al., 2016). Inflammation has been shown to contribute to the onset and progression of AD (Chen and Yu, 2023; Griffin, 2013; Lopez-Rodriguez et al., 2021). Chronic peripheral inflammation caused microglial activation and subsequently induced the formation of A1 astrocytes, contributing to the death of neurons and oligodendrocytes in neurodegenerative diseases (Liddelow et al., 2017). Elevated levels of pro-inflammatory factors were observed in both brain tissue and serum of AD patients, including IL-1α, IL-1β, IL-6, and TNF-α. In addition, elevated serum TNF-α, IL-6 levels directly linked to neuropsychiatric features in AD (Jorfi et al., 2023; Rubio-Perez and Morillas-Ruiz, 2012). Both IL-1α and IL-1β gene polymorphisms were significantly related to an increased AD incidence (Beydoun et al., 2019). Inflammatory factors like TNF-α and IL-8 significantly predicted a one-year decline in executive function in AD patients. And higher IL-6 levels were predictive of subsequent conversion to mild cognitive impairment (MCI) and a greater accumulation of Aβ over a two-year period (Bawa et al., 2020; Oberlin et al., 2021). In addition, elevated serum levels of VCAM-1 were observed in AD patients than healthy controls (Zuliani et al., 2008). AD severity, macro- and micro-structural changes to white matter, poor short-term memory and visuospatial function were all linked to VCAM-1 levels (Huang et al., 2015). The above findings suggested that VVs might potentially contribute to the initiation and progression of AD via upregulating the levels of inflammatory factors.

Another possible explanation is that VVs might be associated with AD risk via affecting circulatory system diseases. VVs are a sign of chronic venous insufficiency. It has been reported that chronic venous insufficiency was associated with cardiovascular diseases (Auzky et al., 2011). The presence of VVs were related to an increased occurrence of cardiovascular events and mortality (Wu et al., 2020). Furthermore, VVs might be related to peripheral artery disease (Chang et al., 2018). Several cross-sectional studies have shown that cardiovascular diseases and peripheral artery disease were linked to an increased incidence of AD (Pasqualetti et al., 2022; Skoog et al., 1999). Taken together, the presence of VVs might elevate the AD risk via affecting circulatory system diseases.

In our subgroup analysis stratified by age, VVs were significantly correlated with CSF AD biomarkers in the mid-life subgroup, while such correlations were not evident in the late-life subgroup. The specific reasons for this age-related difference were not fully understood, but the increased venous pressure caused by VVs was similar to the condition of increased arterial pressure (Arase et al., 2019), which made us think of hypertension. Hypertension has been demonstrated to be an age-specific risk factor for both cognitive impairment and AD, with significant associations in mid-life and less significant associations in late-life (Hu et al., 2022; Livingston et al., 2020; Ou et al., 2020). Combining this evidence and our finding, it can be inferred that mid-life patients with vascular diseases characterized by elevated pulse pressure, whether hypertension or VVs, were more likely to have pathological changes of AD. It is suggested that VVs may be a potential predictor of the future AD risk, particularly in mid-life patients. We also found that VVs were significantly correlated with CSF Aβ_42_, CSF p-tau, Aβ_42_/Aβ_40_, t-tau/Aβ_42_ and p-tau/Aβ_42_ ratio in female participants, whereas they were only correlated with CSF Aβ_42_/Aβ_40_ ratio in male participants. This finding should be interpreted with caution since it might be confounded by the greater incidences of both VVs and AD in females (Alzheimers Dementia, 2023; Hamdan, 2012). Besides, our study found that VVs were significantly associated with CSF AD biomarkers among subjects with lower education levels, while such an association was not observed in those with higher education level. This finding may be related to the modulatory role of education level in AD pathology. For example, a study has found that, with increasing age, individuals with higher levels of education (indicative of greater cognitive reserve) exhibit a slower age-related increase in CSF t-tau and p-tau levels (Almeida et al., 2015). Another study has found that among individuals with higher education levels, greater midlife cognitive activity is associated with lower Aβ deposition in *APOE-ε4* carriers (Vemuri et al., 2016). These findings suggest that higher education levels may influence the trajectory of AD core biomarkers, thereby attenuating the observed association between VVs and AD biomarkers.

Prior research has indicated that the *APOE-ε4* gene presence exacerbates neurodegeneration, while the absence of *APOE-ε4* has a strong protective effect on neurons (Shi et al., 2017). However, our results showed that *APOE-ε4* non-carriers had more significant associations between VVs and CSF AD biomarkers compared to *APOE-ε4* carriers. In *APOE-ε4* carriers, VVs were associated only with CSF Aβ_42_ and p-tau/Aβ_42_ ratio. Nevertheless, the comparatively small proportion of *APOE-ε4* carriers (15.5%) in our study could have led to false-negative results. Direct clinical evidence regarding the relationship between VVs and *APOE-ε4* carrier status is currently lacking, and studies with larger sample sizes as well as longer follow-up durations are essential in the future.

Our study demonstrated that VVs were associated with both Aβ pathology and p-tau pathology. Causal mediation analysis demonstrated that the link between VVs and p-tau pathology was partially mediated by CSF Aβ_42_/Aβ_40_ ratio. This result could be explained by amyloid cascade hypothesis, where excessive accumulation of toxic Aβ is considered as the upstream initiator of a cascade of events leading to neurodegeneration (van der Kant et al., 2020; Welikovitch et al., 2018).

The reliability of our results is reinforced by several strengths. Firstly, our results were applied to the preclinical phase of AD by using non-demented participants, and therefore we have avoided potential confounding bias from complex AD conditions and poor ability to perform activities of daily living. Secondly, this study stood as the first and largest to date to investigate the relationship between VVs and CSF AD biomarkers. However, this study also had several limitations that cannot be overlooked. Firstly, our study was limited to a cross-sectional study, preventing the establishment of a cause-and-effect relationship between VVs and CSF AD biomarkers. Replication of our findings in larger-scale longitudinal cohorts is crucial for future validation. Secondly, the lack of relevant data restricted our exploration of inflammatory factors, which might be a mechanism that underlies the association between VVs and the development of AD. Finally, given that the association between VVs and AD pathology may be better reflected by brain imaging data, future prospective neuroimaging cohorts would make it easier to validate the role of VVs.

## Conclusion

In conclusion, the connections between VVs and CSF AD biomarkers were demonstrated in our study. It provided novel insights into the mechanisms underlying the association between VVs and AD. Early intervention for patients with VVs, especially in mid-life people, females and less-educated individuals, are recommended in AD prevention.

## Data Availability

The datasets utilized in the study are not publicly available due to privacy or ethical restrictions. Requests to access this data should be directed to the corresponding authors.
