# Supplementary material for: Associations of varicose veins with cerebrospinal fluid biomarkers of Alzheimer’s disease pathologies in adults without dementia: the CABLE study
Source: Front Aging Neurosci. 2025 Mar 4;17:1502154. doi: 10.3389/fnagi.2025.1502154 (PMC11913850; doi:10.3389/fnagi.2025.1502154)
Supplement: Supplementary file 1 [file Table_1.docx]

**Associations of Varicose Veins with Cerebrospinal Fluid Biomarkers of Alzheimer’s Disease Pathologies in Adults without Dementia: the CABLE Study**

[***Supplementary Table 1 2***](#_Toc151295941)

**Supplementary Table 1. Associations of VVs with CSF AD biomarkers in subgroups.**

Associations of VVs with CSF AD biomarkers in subgroups

|  | Aβ42 | | Aβ40 | | t-tau | | p-tau | | Aβ42/Aβ40 | | t-tau/Aβ42 | | p-tau/Aβ42 | |
| --- | --- | --- | --- | --- | --- | --- | --- | --- | --- | --- | --- | --- | --- | --- |
|  | β | p | β | p | β | p | β | p | β | p | β | p | β | p |
| Mid-life† | -0.1531 | 0.0755 | 0.1843 | **0.0364** | 0.0533 | 0.5237 | 0.1926 | **0.0240** | -0.2702 | **0.0019** | 0.1685 | **0.0482** | 0.2477 | **0.0039** |
| Late-life† | -0.2006 | 0.2041 | 0.1028 | 0.5012 | 0.1049 | 0.4995 | 0.1417 | 0.3556 | -0.2915 | 0.0599 | 0.2465 | 0.1190 | 0.2582 | 0.1020 |
| Female# | -0.2284 | **0.0464** | 0.1531 | 0.1699 | 0.1266 | 0.2470 | 0.2189 | **0.0498** | -0.3385 | **0.0030** | 0.2869 | **0.0122** | 0.3222 | **0.0049** |
| Male# | -0.0931 | 0.3574 | 0.1889 | 0.0646 | 0.0490 | 0.6080 | 0.1700 | 0.0779 | -0.2169 | **0.0323** | 0.1084 | 0.2774 | 0.1829 | 0.0698 |
| *APOE* (-)* | -0.0970 | 0.2463 | 0.2721 | **0.0011** | 0.1334 | 0.0905 | 0.2543 | **0.0016** | -0.2906 | **0.0005** | 0.1627 | **0.0487** | 0.2171 | **0.0090** |
| *APOE* (+)* | -0.4629 | **0.0086** | -0.3325 | 0.0587 | -0.1562 | 0.3680 | -0.1015 | 0.5430 | -0.1997 | 0.2530 | 0.3465 | 0.0510 | 0.4219 | **0.0183** |
| Less-edu‡ | -0.1667 | 0.0978 | 0.2247 | **0.0224** | 0.2220 | **0.0187** | 0.2700 | **0.0051** | -0.3294 | **0.0009** | 0.2925 | **0.0033** | 0.2963 | **0.0032** |
| Higher-edu‡ | -0.1387 | 0.2269 | 0.1060 | 0.3686 | -0.0982 | 0.3780 | 0.0984 | 0.3790 | -0.2000 | 0.0883 | 0.0513 | 0.6535 | 0.1837 | 0.1085 |

†Adjusting for gender, education levels, and *APOE-ε4* status.

#Adjusting for age, education levels, and *APOE-ε4* status.

*Adjusting for age, gender, and education levels.

‡Adjusting for age, gender and *APOE-ε4* status.

Abbreviations: VVs, varicose veins; CSF, cerebrospinal fluid; AD, Alzheimer’s disease; Aβ, amyloid-β; t-tau, total tau; p-tau, phosphorylated tau; *APOE-ε4*, apolipoprotein E genotype ε4, Less-edu, less-educated; Higher-edu, higher-educated.
